# A large normative connectome for exploring the tractographic correlates of focal brain interventions

**DOI:** 10.1038/s41597-024-03197-0

**Published:** 2024-04-08

**Authors:** Gavin J. B. Elias, Jürgen Germann, Suresh E. Joel, Ningfei Li, Andreas Horn, Alexandre Boutet, Andres M. Lozano

**Affiliations:** 1 Division of Neurosurgery, Department of Surgery, University Health Network and University of Toronto, Toronto, Canada; 2 Krembil Research Institute, University of Toronto, Toronto, Canada; 3 Center for Advancing Neurotechnological Innovation to Application (CRANIA), University Health Network, Toronto, Canada; 4 GE Global Research, Bangalore, India; 5 Department of Neurology, Charité-Universitätsmedizin Berlin, Berlin, Germany; 6 Einstein Center for Neurosciences Berlin, Charité-Universitätsmedizin Berlin, Berlin, Germany; 7 Center for Brain Circuit Therapeutics, Department of Neurology, Brigham & Women’s Hospital, Harvard Medical School, Boston, USA; 8 Department of Neurosurgery, Massachusetts General Hospital, Harvard Medical School, Boston, USA; 9 Joint Department of Medical Imaging, University of Toronto, Toronto, Canada

**Keywords:** Brain injuries, Neural circuits, Brain, Biomarkers

## Abstract

Diffusion-weighted MRI (dMRI) is a widely used neuroimaging modality that permits the *in vivo* exploration of white matter connections in the human brain. Normative structural connectomics – the application of large-scale, group-derived dMRI datasets to out-of-sample cohorts – have increasingly been leveraged to study the network correlates of focal brain interventions, insults, and other regions-of-interest (ROIs). Here, we provide a normative, whole-brain connectome in MNI space that enables researchers to interrogate fiber streamlines that are likely perturbed by given ROIs, even in the absence of subject-specific dMRI data. Assembled from multi-shell dMRI data of 985 healthy Human Connectome Project subjects using generalized Q-sampling imaging and multispectral normalization techniques, this connectome comprises ~12 million unique streamlines, the largest to date. It has already been utilized in at least 18 peer-reviewed publications, most frequently in the context of neuromodulatory interventions like deep brain stimulation and focused ultrasound. Now publicly available, this connectome will constitute a useful tool for understanding the wider impact of focal brain perturbations on white matter architecture going forward.

## Background & Summary

The brain networks underlying healthy function and disease have long been a major focus of neuroscientific research. Many of the earliest insights into this topic came from focal lesion studies and cortical stimulation work, including famous studies by pioneers like Broca^1^ and Penfield^2^. While that body of work remains relevant to this day^3,4^, subsequent studies have built on this foundation using newer non-invasive neuroimaging and electrophysiological techniques, such as PET/SPECT^5^, EEG^6^, and MRI^7^. Most recently, further MRI advances have facilitated more direct *in vivo* interrogations of brain connectivity – both in circumscribed areas of interest, and across the whole brain (‘connectomics’). This line of inquiry has primarily used two MRI techniques: resting state functional MRI (rsfMRI), which relies on BOLD signal fluctuations to infer region-to-region crosstalk (i.e., the coordinated workings of the brain)^8^, and diffusion-weighted MRI (dMRI), which approximates white matter connections in the brain based on the directionality and anisotropy of water diffusion^9^. Importantly, both techniques emphasize the interconnectedness of disparate brain regions and have been widely applied to neuromodulation research in particular, offering a means to explore how focal interventions such as lesions or electrical stimulation might impact distributed networks^10^. While these MRI sequences can be acquired in individual subjects, they can also be utilized to build ‘normative connectomes’: group-level aggregates of rsfMRI or dMRI scans obtained from a large number of other subjects. Normative connectomes have been described as generalized ‘wiring diagrams’ of the human brain and offer the obvious advantage that they can be applied to any cohort of interest, not just those in whom dMRI or rsfMRI sequences have been acquired^10^. This is particularly relevant in the case of patients undergoing procedures like deep brain stimulation (DBS), in whom these sequences are often not routinely obtained and may be contraindicated due to issues like movement artifacts and hardware safety^11,12^. Normative connectomes may also play a special role in understanding how spontaneously occurring focal insults (e.g., in the context of stroke) disrupt the normal organization of the brain^13^ and how this may produce various symptoms. In particular, numerous studies have employed normative connectivity mapping to probe for distributed networks that may underpin post-stroke phenomena such as central pain^14^, parkinsonism^15^, and depression^16^. In each case, the connectome is typically ‘seeded’ using a region-of-interest (ROI), which represents the site of network perturbation, yielding a set of fiber streamlines (structural) or correlated brain areas (functional) that would tend to be impacted by this perturbation in the typical individual. Normative connectomics can also be used to augment more traditional neuroimaging analyses, such as by calculating the connectivity patterns of areas where differences or changes in brain structure or activity/metabolism were detected^17,18^.

Normative dMRI-based connectomes (i.e., ‘structural’ normative connectomes) in particular have seen increasingly widespread use as a tool for exploring the network-level correlates of neuromodulatory interventions (see *Elias et al. 2022* for review)^19^. This prevalence may in part relate to the fact that the white matter streamlines identified by structural connectomics offer a more tangible, readily visualized fiducial with which to guide procedural targeting and (in the case of DBS) post-operative refinement of stimulation location through parameter selection. Published neuromodulation studies employing structural connectomic techniques have used a variety of connectome datasets for their analyses. The most frequently used structural connectomes have been assembled from healthy adult MRI scans collected as part of the Human Connectome Project (HCP). HCP scans are acquired using specialized MR hardware and consequently boast superior signal-to-noise ratios and fidelity than can be achieved at most academic centres^20,21^. The number of HCP subjects sampled to construct these connectomes has also varied; a handful of studies have used larger connectomes compiled from ~400–850 individuals^22,23^, while the majority have utilized connectomes aggregated from 30–40 healthy subjects^24–34^. Other studies have leveraged dMRI scans from patients with the same condition as the population of interest in order to construct ‘disease-specific’ connectomes that might better capture the connectivity differences that likely characterize patients with certain longstanding neurological conditions^35^. To date, this has primarily been attempted in the context of Parkinson’s disease using dMRI scans acquired as part of the Parkinson’s Progression Markers Initiative (PPMI), with structural connectomes ranging from ~40 to ~90 subjects in size^31,33,36–42^.

The current study presents and describes a newer HCP-derived, MNI-space structural connectome that has been assembled from the multi-shell dMRI scans of 985 healthy young adults and comprises ~12 million fiber streamlines – the largest connectome of its kind yet described. While made publicly available for the first time here, this connectome has in fact been employed for at least 18 published articles on topics such as DBS for varied neurological and psychiatric conditions^14,17,18,43–51^, focused ultrasound (fUS) for essential tremor^52–54^, naturally occurring or surgically created lesions resulting in neuropsychiatric sequelae^14,55^, and exploratory papers examining future applications of connectomics^56,57^. The connectome comprises a whole-brain tractogram that can be seeded with ROIs (e.g., DBS activation volumes or brain lesions) to identify and output streamlines that traverse the seed regions. This in turn facilitates exploration of the wider connectivity profile of focal brain interventions or injuries and – when paired with clinical/behavioural data – also enables streamline-level investigations into the relationship between white matter engagement and clinical outcome. These kinds of analysis have so far been conducted primarily – although not exclusively – using DBS activation volumes, but can be similarly performed using ROIs ranging from stroke lesions or non-invasive stimulation activation fields through to loci of group-wise volumetric/functional neuroimaging differences. Here, we detail the steps used to construct this normative connectome, describe the various freely available neuroimaging files and scripts that facilitate its use, and provide evidence to validate its anatomical basis and applicability to neuromodulatory research.

## Methods

### Data acquisition

This connectome was assembled using MRI data from healthy young adults who were scanned as part of the Human Connectome Project (HCP) S1200 subject release (https://www.humanconnectome.org/study/hcp-young-adult/document/1200-subjects-data-release). Specifically, T1-weighted, T2-weighted, and multi-shell dMRI scans were downloaded from the S1200 subject release repository. A total of 1065 individual subjects’ scans were initially downloaded, which yielded usable data (i.e., one of each of the aforementioned sequences) from 1000 subjects after incomplete and corrupted files were discarded. Of this 1000-subject-strong cohort, 538 subjects (53.8%) were female. By age range at the time of scanning, 217 subjects (21.7%) were 22–25 years of age, 433 (43.3%) were 26–30 years of age, 341 (34.1%) were 31–35 years of age, and 9 (0.9%) were 36 or older.

All MR images had been obtained using customized 3.0 Tesla Connectome Skyra scanners with high-end gradient coils. The acquisition parameters for each sampled sequence were as follows: i) T1-weighted scan (3D MPRAGE): TR = 2400 ms; TE = 2.14 ms; TI = 1000 ms; flip angle = 8 deg; FoV = 224 × 224 mm; voxel size = 0.7 mm isotropic; ii) T2-weighted scan (3D T2-SPACE): TR = 3200 ms; TE = 565 ms; flip angle = variable; FoV = 224 × 224 mm; voxel size = 0.7 mm isotropic; iii) multi-shell dMRI: TR = 5520 ms; TE = 89.5 ms; flip angle = 78 deg; FoV = 210 × 180 mm; voxel size = 1.25 mm isotropic; number of gradient tables = 3; number of b0 acquisitions = 6 (per gradient table); number of diffusion weighting directions = 90 (per gradient table); b-values = 1000, 2000, and 3000 s/mm^2^. Previous work has indicated that multi-shell dMRI data (i.e., dMRI scans acquired with multiple b-values) confers greater sensitivity to non-dominant fiber populations^58^. All imaging files had already been subjected to the HCP Minimal Preprocessing Pipeline, which removed spatial/artefact distortion, performed cross-modal registration, and aligned (i.e., rigidly registered using 6 degrees of freedom) images to MNI152 standard space^21^. With respect to the dMRI scans specifically, this preprocessing pipeline normalized the b0 image intensity across runs and corrected for EPI distortions, eddy-current-induced distortions, gradient-nonlinearities, and subject motion.

### Image normalization

**Fig. 1:**
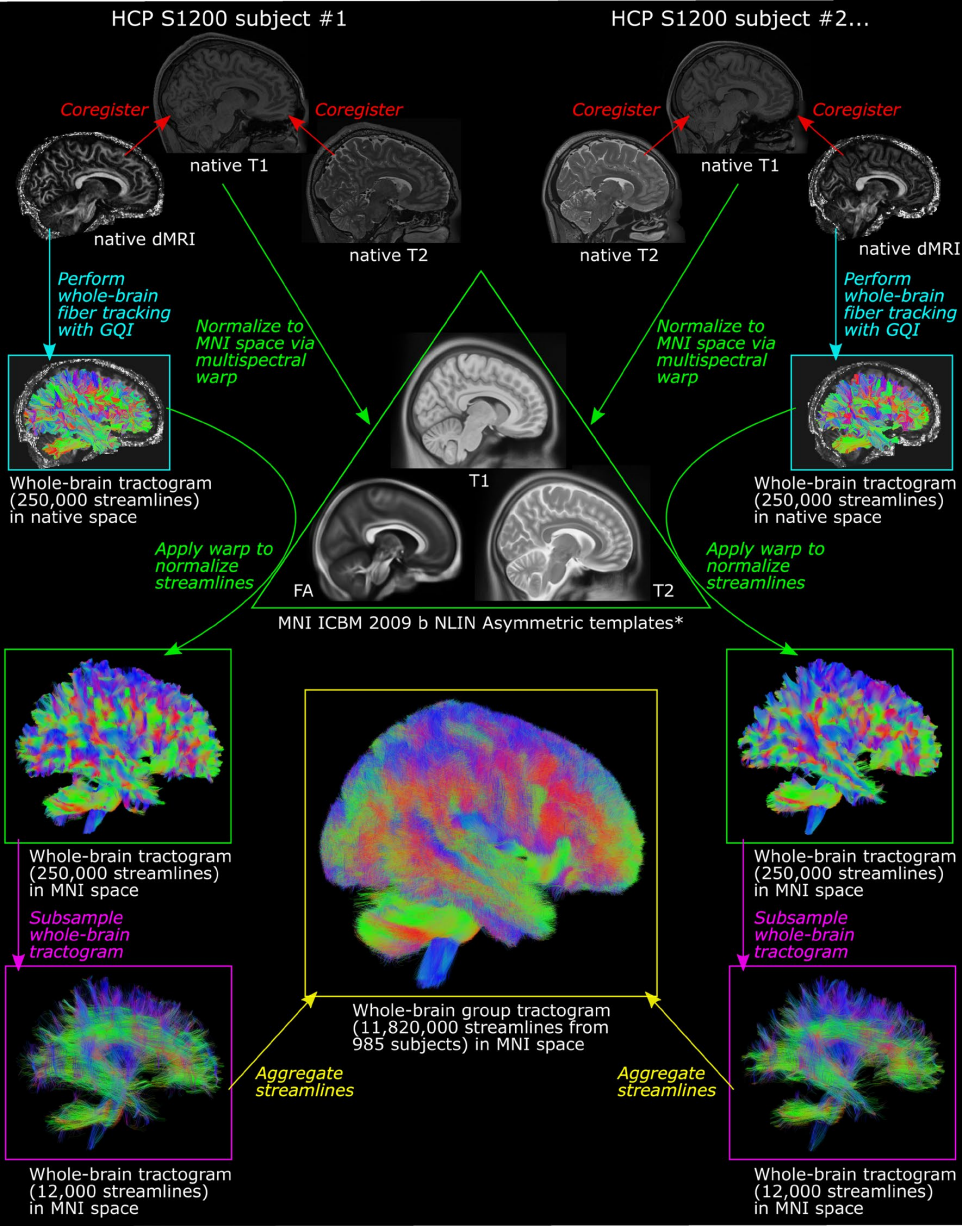
Visual summary of connectome construction pipeline. The major steps (coloured arrows, italicized and coloured text) taken to build the structural connectome are outlined alongside representative brain images from two subjects. First, the native T1-weighted, T2-weighted, and dMRI acquisitions for each HCP S1200 subject were coregistered (red arrows). Next, all three native scans were nonlinearly normalized to MNI ICBM 2009b NLIN Asymmetric space using a multispectral warp approach (green arrows). (*This was an iterative process and involved the creation of a new, 1000-subject FA template in MNI ICBM 2009b NLIN Asymmetric space – please see *Image normalization* section of Methods for further information). Separately, a whole-brain tractogram (250,000 streamlines) was generated from each patient’s native dMRI data using GQI (turquoise arrows). This tractogram was then normalized to MNI space using the previously obtained subject-specific transform (curved green arrows). Each tractogram was subsampled, randomly selecting 12,000 streamlines from the total streamline count (magenta arrows). Finally, the subsampled streamlines of all 985 HCP subjects were aggregated to form a single, 11,820,000-streamline group tractogram in MNI space. *FA* = fractional anisotropy; *GQI* = generalized Q-sampling imaging; *HCP* = Human Connectome Project; *MNI* = Montreal Neurological Institute.

The downloaded HCP data were then processed using Lead-Connectome (https://www.lead-dbs.org/about/lead-connectome/), a matlab-based toolbox that is freely available as part of the Lead-DBS software package (https://www.lead-dbs.org/). We chose to use this software primarily on the basis of ease of use; it constituted a convenient wrap-around package that integrated well with many of the other tools necessary for this analysis. The connectome construction pipeline is summarized in Fig. 1. First, an initial round of multispectral normalization to MNI space was performed. This entailed (i) linearly coregistering each subject’s fractional anisotropy (FA) and T2-weighted scans to their T1-weighted scan using SPM (https://www.fil.ion.ucl.ac.uk/spm/software/spm12/)^59^; (ii) nonlinearly normalizing all three image types in a multispectral fashion to Montreal Neurological Institute (MNI ICBM 2009b NLIN Asymmetric) standard space using the ‘effective low variance’ preset (as implemented in Lead-DBS) of the ANTs SyN algorithm (http://stnava.github.io/ANTs/)^60,61^. FA images were derived from the originally downloaded dMRI files using code originally published in the DTI and Fiber Tracking Matlab library (https://www.mathworks.com/matlabcentral/fileexchange/21130-dti-and-fiber-tracking)^62^ and implemented in Lead-DBS (https://github.com/netstim/leaddbs/blob/master/ext_libs/ftracking/ea_DTI.m). For the initial normalization step, the contribution of the FA images to the multispectral warp was disabled; this was to avoid problems associated with attempting to register the high-resolution HCP FA acquisitions to the pre-existing FA MNI template (an adapted version of the 2 × 2 × 2 FMRIB 58 template that had been resampled to 2009b NLIN Asymmetric space). The adequacy of coregistration and normalization for each of the three images was determined through visual inspection and any poorly registered/normalized files were discarded. Specifically, we manually assessed Lead-DBS-generated quality control images for each transformation, verifying that the contours of prominent brain structures such as the cerebellum, brainstem, thalamus, and cerebral lobes were closely aligned (i.e., within a few millimetres) between the coregistered/normalized source image and the target image. No subjects were excluded at this stage. Next, the 1000 normalized FA images were averaged using FSL tools (FMRIB Software Library) to create a new, high-resolution FA template in MNI 2009b NLIN Asymmetric space. A second round of multispectral normalization to MNI space was then performed using the newly created high-resolution FA template. The same ANTs algorithm outlined above was employed but the contribution of the individual FA images to the warp was this time enabled. The results of this step were again visually inspected as described above and 15 subjects were discarded on account of poor normalization outcome (see Supplementary Figure 1 for exemplar quality control images).

### Fiber tracking and connectome construction

Whole-brain fiber tracking was next performed using the dMRI data from the remaining 985 subjects. Of this cohort, 529 subjects (53.7%) were female. By age range at the time of scanning, 212 subjects (21.5%) were 22–25 years of age, 430 (43.7%) were 26–30 years of age, 334 (33.9%) were 31–35 years of age, and 9 (0.9%) were 36 or older. Fiber tracking was performed in the native (i.e., non-normalized) subject dMRI space using generalized Q-sampling imaging (GQI, a model-free deterministic tractography method) as implemented in DSI Studio (http://dsi-studio.labsolver.org)^63^. GQI has been shown to better resolve crossing fibers compared to more traditional techniques such as diffusion tensor imaging^64^.

In each subject, 250,000 streamlines (minimum length = 10 mm, maximum length = 500 mm) were sampled from across the whole brain, as defined by a white matter mask based on the coregistered T2-weighted scan. Tracking parameters included a step size of 0.46875 mm and an angular threshold of 60°. The anisotropy threshold was automatically determined by DSI Studio. Utilizing the transforms described in the previous section, each subject’s fiber streamlines were then warped into MNI 2009b NLIN Asymmetric space using previously described methods implemented through the Lead-DBS software package^65,66^. After visually inspecting the fiber tracking and fiber normalization results (no unsatisfactory results were observed), the normalized fiber streamlines from each subject were aggregated into a single template using Lead-Group Connectome. Specifically, 12,000 streamlines were randomly subsampled from each subject’s previously identified 250,000 streamlines; these were then combined to create a final group tractogram comprising 11,820,000 unique streamlines.

## Data Records

### Data records as a contribution

The data and scripts described in this data publication are freely available on FigShare^67^.

This work contributes data records that permit the use of our HCP-derived whole-brain structural connectome (nicknamed ‘dTOR-985’ [Toronto 985-subject diffusion-weighted MRI connectone]) for neuroimaging analyses at different resolutions. First, the complete connectome itself is provided in the form of a matlab-readable file (‘dTOR_985.mat’). A version of the full connectome that can be directly viewed and manipulated using streamline viewing/editing programs like MI-Brain (https://github.com/imeka/mi-brain) or TrackVis (https://trackvis.org) – i.e., a whole-brain tractogram file comprising 11,820,000 streamlines (“dTOR_full_tractogram.trk”) – is provided as well. Three voxel-resampled connectome files are also supplied in matlab-readable format: one in 0.5 mm resolution (‘dTOR_fibers_vox_half_mm.mat’), one in 1.0 mm resolution (‘dTOR_fibers_vox_1_mm.mat’), and one in 2.0 mm resolution (‘dTOR_fibers_vox_2_mm.mat’). These files share their resolutions and spacing with the MNI ICBM Asymmetric template brains (https://www.bic.mni.mcgill.ca/ServicesAtlases/ICBM152NLin2009) and contain the ‘fibers_vox’ variable, a 1 × 11,820,000 cell array that – for each streamline in the full connectome – lists the x/y/z coordinates of all voxels within the respective MNI brain template that encompass said streamline. Please note that the voxel-resampled connectome files are provided in zipped format and must be unzipped prior to use.

**Fig. 2:**
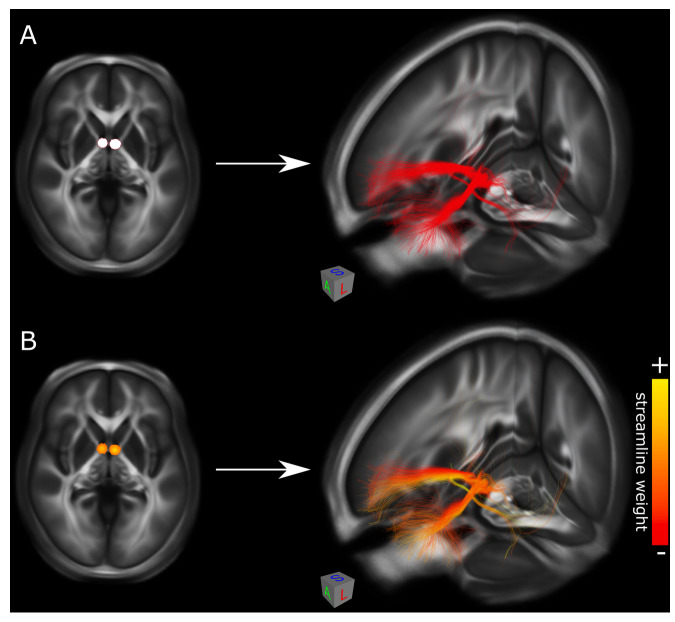
Weighted and unweighted streamline output. Exemplar seeds and streamline outputs are displayed on the backdrop of a high-resolution FA template in MNI space. (**A**) Seeding the structural connectome with a binary ROI (e.g., a DBS activation volume/volume of tissue activated; left image) generates unweighted streamlines that intersect the ROI (right image). (**B**) Using a non-binary ROI (e.g., a DBS e-field; left image) instead generates weighted streamlines whose value reflects that of the highest-value ROI voxel they intersect (right image). *FA* = fractional anisotropy; *DBS* = deep brain stimulation; *ROI* = region-of-interest.

Two self-contained custom-built matlab scripts are also provided: the ‘dTOR_compute_fiber_weights.m’ script, and the ‘dTOR_create_trk.m’ script. These scripts are to be run one after the other to identify and output fiber streamlines that overlap a given region-of-interest (ROI). The freely available SPM software package (https://www.fil.ion.ucl.ac.uk/spm/software/spm12/)^59^ must be downloaded and added to the matlab path before use. The first script (‘dTOR_compute_fiber_weights.m’) takes an ROI input file in nifti format and uses this to seed the structural connectome, identifying streamlines that intersect the ROI and ascribing them a certain weight. This weight corresponds to the voxel value of the ROI, which may be either binary or non-binary. If the ROI is binary, all streamlines that intersect it will be given a value of 1. If the ROI is non-binary, streamlines intersecting it will be assigned a value corresponding to the highest-value voxel that they intersect (Fig. 2). This script must be pointed towards one of the voxel-resampled connectome files; the choice of which file to use determines the resolution in which the computation will be performed (note that the ROI file must also match this resolution; this can be achieved by first resampling the ROI file to the appropriate MNI template brain). With respect to specific computational methods, the script first loads the voxel-resampled connectome .mat file. It then reads the header information and voxel values of the input ROI file, creating a 3D array (‘im’) whose values are normalized by the maximum voxel value in the input file. Next, the script creates a vector (‘fibers_wt’, initialized with zeros) with length equal to the number of elements in the voxel-resampled connectome file’s ‘fibers_vox’ variable (i.e., the total number of streamlines in the connectome). It subsequently initiates a loop to iterate through each streamline in ‘fibers_vox’; within this loop, another loop iterates through each all of the voxels that encompass said streamline, checking if these voxels also have a non-zero value within the ROI ‘im’ array and assigning this non-zero value to the corresponding element in the ‘fibers_wt’ vector in this circumstance. By the end of these loops, the ‘fibers_wt’ vector thus contains information about ROI overlap with each streamline in the connectome, denoting non-overlap with a 0 and overlap with a non-zero value. This information is saved and output as a .mat file that may be opened and processed using software like matlab or R to conduct statistical analyses (e.g., to elucidate ‘discriminative streamlines’ using streamline-level t-tests or to determine which streamlines are common to all or most patients in a given cohort).

To convert the information saved in the matlab data file to a viewable form, the second script (‘dTOR_create_trk.m’) must be run. This script takes the .mat file created by the first script (or another .mat file, such as one generated through statistical analysis) as an input and writes out a tractogram file (.trk) that can be opened in a streamline viewing/editing software such as MI-Brain (https://github.com/imeka/mi-brain) or TrackVis (https://trackvis.org). It must also be pointed towards the complete, non-resampled connectome file (‘dTOR_985.mat’), as this file contains the ‘fibers’ variable from which the script extracts the fiber points information necessary to reconstruct the streamlines specified in the input .mat file. The dTOR_create_trk.m script also allows the user to set thresholds for which streamlines to generate based on streamline weight, and to specify the colour scheme used for visualizing these weights.

This work also provides files that permit the user to conduct an equivalent streamline analysis using the Lead-DBS software package (https://www.lead-dbs.org/). Detailed instructions on how to use these files as part of the Lead-DBS pipeline can be found on the corresponding website. A ‘full’ version of our structural connectome for use in Lead-DBS is provided, as are ‘half-scale’ (aggregating 6000 streamlines from each HCP subject), and ‘quarter-scale’ (aggregating 3000 streamlines from each HCP subject) versions.

Finally, this work also contributes a newly computed, high-resolution (voxel size: 0.5 × 0.5 × 0.5 mm) FA template in MNI 2009b NLIN Asymmetric space, created as part of our connectome construction pipeline by averaging the FA images of 1000 healthy young adults (https://www.humanconnectome.org/study/hcp-young-adult/document/1200-subjects-data-release). This file (FA_template_1000_HCP.nii.gz) may have utility as a registration template for high-resolution dMRI data, as was the case for our connectome construction pipeline. It is provided in nifti format (nii.gz) and can be readily visualized with standard software tools such as FSLeyes (https://fsl.fmrib.ox.ac.uk/fsl/fslwiki/FSLeyes) and Display (https://www.bic.mni.mcgill.ca/software/Display/Display.html).

### Original datasets used

The original data used for connectome construction were provided by the Human Connectome Project, WU-Minn Consortium (Principal Investigators: David Van Essen and Kamil Ugurbil; 1U54MH091657) funded by the 16 NIH Institutes and Centers that support the NIH Blueprint for Neuroscience Research; and by the McDonnell Center for Systems Neuroscience at Washington University. These data are available from https://www.humanconnectome.org/study/hcp-young-adult/document/1200-subjects-data-release.

## Technical Validation

### Canonical white matter tract reconstruction

**Fig. 3:**
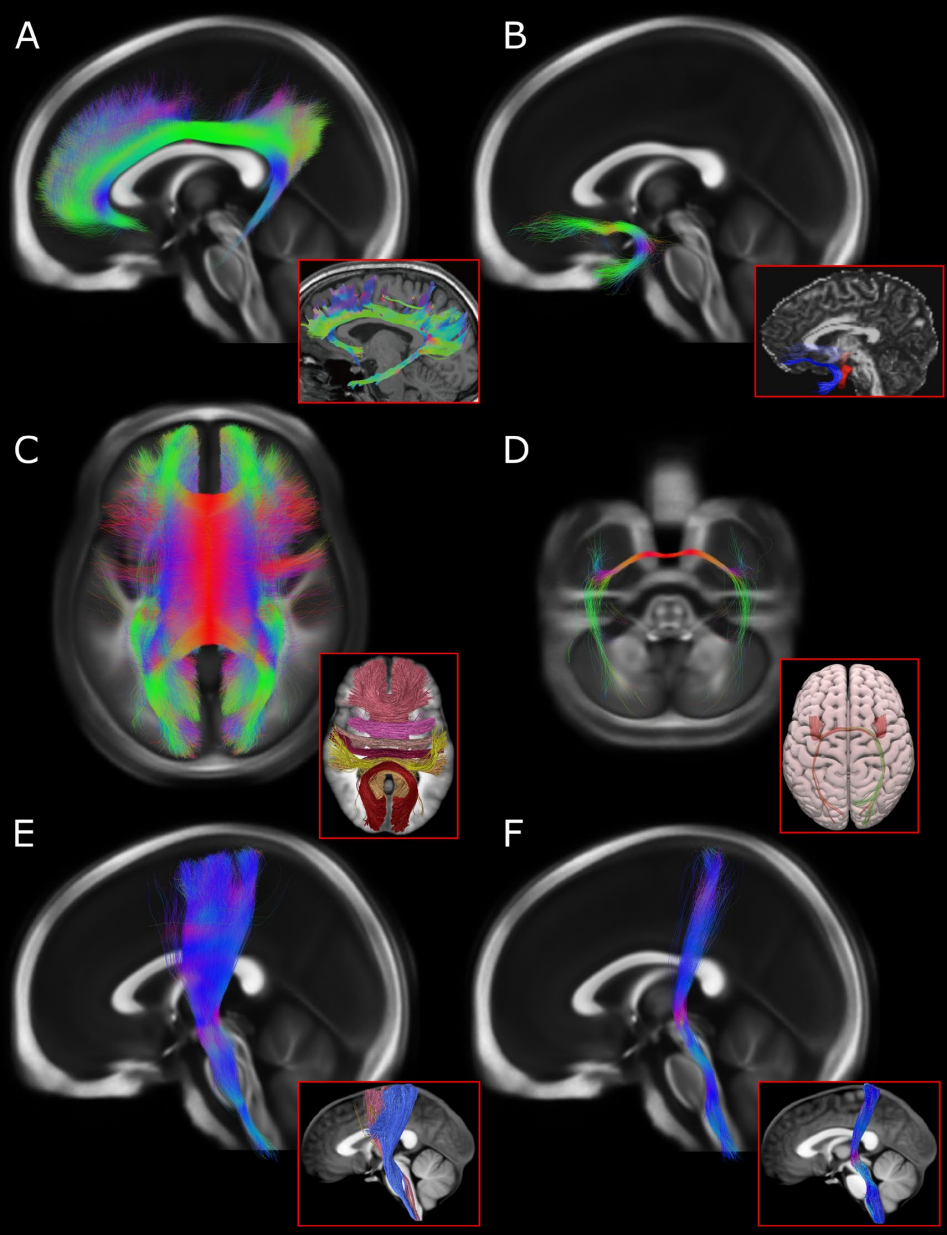
Virtual dissection of canonical white matter tracts. Exemplar association, commissural, and projection bundles are delineated from the 985-HCP subject connectome and compared with literature-derived equivalents (inset panels framed with red borders). Each bundle is displayed in three-dimensions against a backdrop of the 1000-HCP subject fractional anisotropy template in MNI ICBM 2009b NLIN Asymmetric space. For display purposes, only 20% of structural connectome streamlines are visualized. (**A**) The left cingulum bundle (midsagittal view) is compared to an equivalent depicted in Wu *et al*.^70^. (**B**) The left uncinate fasciculus (midsagittal view) is shown alongside an equivalent (blue) from Baur *et al*.^71^. (**C**) The full corpus callosum (superior view) is exhibited alongside a a parcellated version depicted in Radwan *et al*.^69^. (**D**) The anterior commissure (superior view) is shown next to a reconstruction by Radwan *et al*.^69^. (**E**) The left corticospinal tract (midsagittal view) is compared to an equivalent (blue) from Radwan *et al*.^69^. (**F**) The left medial lemniscus (midsagittal view) is displayed alongside an equivalent from Radwan *et al*.^69^. The literature-derived tracts shown in (**A,B**) represent the dMRI data of a single subject, while those shown in (**C**–**F**) represent aggregate data from 20 subjects. All reference images are adapted with minor edits from their original publications under a Creative Commons Attribution License (CC BY). *HCP* = Human Connectome Project.

To demonstrate that our structural connectome can be used to conduct valid, anatomically sound streamline-based analyses, we performed virtual dissections to isolate major canonical white matter tracts. This was accomplished by generating ROIs in MNI space – informed by previously published dMRI virtual dissection atlases^68,69^ – and using these to seed the connectome. Exemplars were created in this way for association (left cingulum bundle and left uncinate fasciculus), commissural (corpus callosum and anterior commissure), and projection pathways (left corticospinal tract and ascending somatosensory fibers of the left medial lemniscus). Each of these reconstructed tracts was consistent with known anatomy and appeared similar to white matter tract bundles featured in previously published dMRI studies (Fig. 3)^69–71^. We also performed a quantitative comparison of these same exemplar tracts with equivalent white matter bundle labels sourced from the publicly available IIT Human Brain Atlas (https://www.nitrc.org/projects/iit/), which is derived from 72 young (age 20–40), healthy subjects^72,73^. To do so, we obtained binary maps of our tracts and evaluated their voxelwise overlap with the IIT bundle labels, thresholding these labels at 5% and 10% of their maximum streamline density. In five out of six cases, our exemplar tracts captured a substantial portion of the 5%-thresholded IIT bundle voxels (left cingulum: 88%; left uncinate: 40%; corpus callosum: 82%; anterior commissure: 54%; left corticospinal tract: 73%) and an even larger share of the 10%-thresholded bundles (left cingulum: 94%; left uncinate: 56%; corpus callosum: 90%; anterior commissure: 61%; left corticospinal tract: 82%). Our left medial lemniscus tract captured only 18% and 25% of voxels belonging to the 5%- and 10%-thresholded equivalent IIT bundle, respectively, although this IIT bundle contained numerous streamlines running within the cerebral peduncle that are not conventionally assigned to the medial lemniscus^74,75^.

### Effect of differing resolutions on streamline sampling

**Fig. 4:**
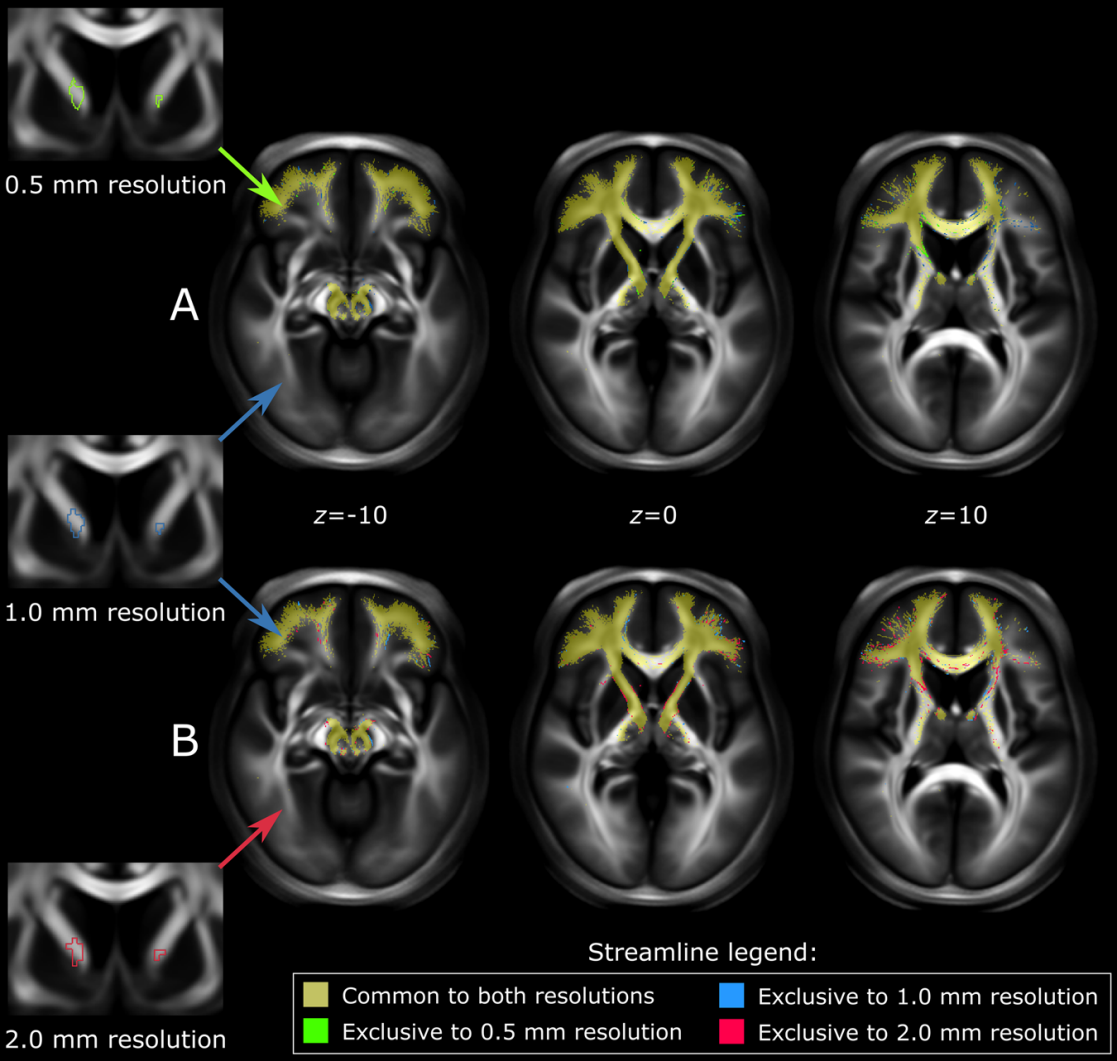
Effect of resolution on streamline sampling output. An ROI in the bilateral internal capsule is used to seed the structural connectome at three different resolutions (0.5 mm – green outline, 1.0 mm – blue outline, 2.0 mm – red outline). Streamline output generated at each resolution is shown as voxelwise binary maps. Images of the ROI and output are visualized on coronal and axial slices, respectively, of a high-resolution FA template in MNI space. Overall, the number and location of streamlines sampled from the structural connectome were similar when compared between (**A**) 0.5 mm and 1.0 mm resolution conditions; (**B**) 1.0 mm and 2.0 mm conditions. By binary map volume, differences between 0.5 mm and 1.0 mm resolution output amounted to 4.8% of the total 0.5 mm resolution output. Differences between 1.0 and 2.0 mm resolution output amounted to 8.7% of the total 1.0 mm resolution output. *FA* = fractional anisotropy; *MNI* = Montreal Neurological Institute.

To clarify the impact that resolution has on fiber tract sampling, we seeded the structural connectome with an exemplar ROI (an ablative fUS lesion within the anterior limb of internal capsule, sourced from the OCD cohort described in Germann *et al*.^52^) three times: once at 0.5 mm resolution, once at 1.0 mm resolution, and once at 2.0 mm resolution. The streamlines generated in each case were similar both in total count (0.5 mm: 68,799 streamlines, 1.0 mm: 70,298 streamlines, 2.0 mm: 71,723 streamlines) and general course (Fig. 4). However, differences were evident when binary maps of each streamline output were compared in a voxelwise fashion. Compared to the 0.5 mm resolution streamline output, the 1.0 mm resolution output differed by 4.8% in terms of volume (i.e., gained or lost streamline-containing voxels). The difference between the 1.0 mm and 2.0 mm resolution streamline output was 8.7% by volume relative to the 1.0 mm output. These results indicate that the connectome is likely suitable for use at lower resolutions, although some variability in streamline output will occur.

### Prior use in neuromodulation patient cohorts

**Fig. 5:**
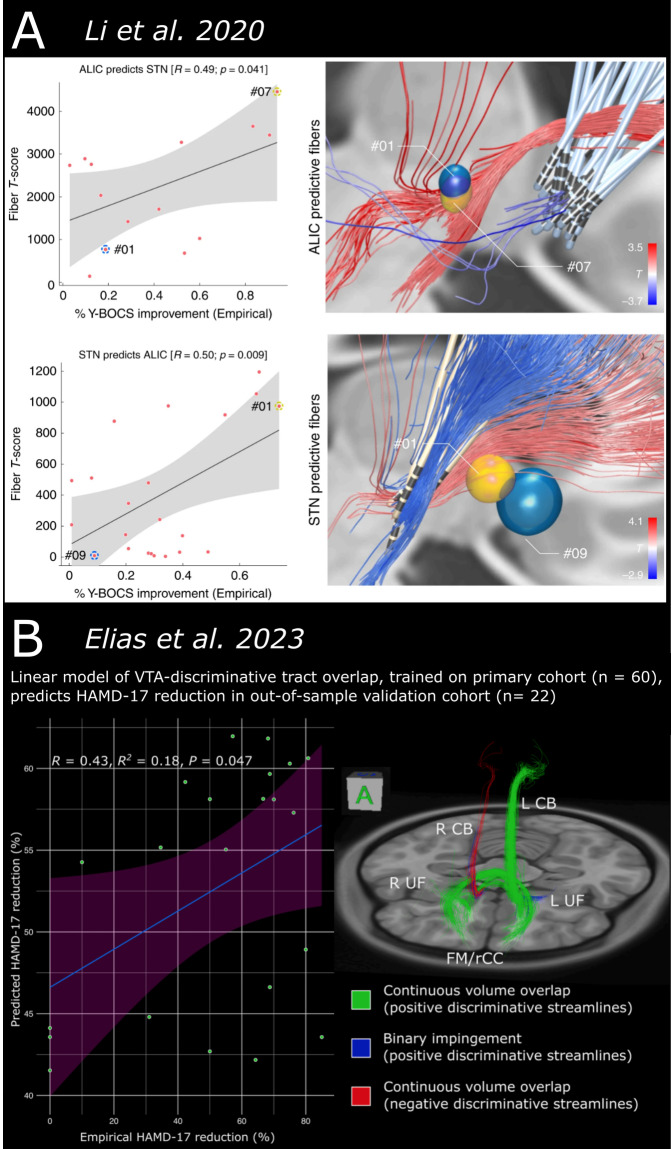
Explaining clinical variance in response to neuromodulatory interventions with structural connectome output. The 985-HCP subject structural connectome has been used in prior publications to explain clinical variance in out-of-sample cohorts. (**A**) Li *et al*.^45^ employed a ‘half-scale’ version of the structural connectome to analyze the relationship between white matter streamline engagement and clinical outcome in OCD patients treated with DBS at various targets. They found that ‘discriminative’ streamlines from a given cohort were able to explain meaningful variance in out-of-sample patients. (**B**) Elias *et al*.^51^ used the full connectome to identify discriminative streamlines in a large cohort of patients treated with SCC-DBS for depression. Overlap with these streamlines was again able to explain meaningful variance in an out-of-sample cohort of SCC-DBS patients. Images are adapted with minor edits from Li *et al*. (panel **A**) and Elias *et al*. (panel **B**) under a Creative Commons Attribution License (CC BY). *DBS* = deep brain stimulation; *HCP* = Human Connectome Project; *OCD* = obsessive-compulsive disorder; *SCC* = subcallosal cingulate area.

As mentioned previously, the structural connectome described here has already been used to explore the network correlates of response to focal neuromodulatory interventions^17,18,43–54^. Two particular published studies – Li *et al*.^45^ and Elias *et al*.^51^ – attest to the potential utility of this connectome in explaining variance in response to DBS (Fig. 5). In Li *et al*., the ‘half-scale’ version of the structural connectome was employed to conduct a streamline-level statistical analysis of fiber engagement in patients treated with DBS of various targets for obsessive-compulsive disorder (OCD). Identifying a bundle of streamlines whose engagement by DBS activation volumes related to clinical improvement (i.e., discriminative streamlines), the authors were able to explain meaningful variance in OCD symptom reduction in out-of-sample cohorts (R = 0.49–0.50, P < 0.05) on the basis of discriminative streamline overlap^45^. More recently, Elias *et al*. leveraged the full-scale structural connectome to perform a similar streamline-level analysis in patients undergoing subcallosal cingulate area DBS (SCC-DBS) for depression. This effort similarly yielded discriminative streamlines that – when segmented into white matter tracts previously implicated in SCC-DBS response^76^ – explained meaningful variance in the improvement of depressive symptomology in an out-of-sample SCC-DBS cohort (R = 0.43, P < 0.05)^51^. These examples speak to the ability of the structural connectome to capture robust clinicoanatomical relationships that might inform neuromodulatory interventions.

### Supplementary information

Supplementary Information

## Data Availability

Our matlab scripts are also accessible on GitHub (https://github.com/Germann-lab/dTOR-985-Connectome.git). The files necessary for using our connectome in the Lead-DBS software package are accessible from Lead-DBS (https://www.lead-dbs.org/).
