# Supplementary material for: A large normative connectome for exploring the tractographic correlates of focal brain interventions
Source: Sci Data. 2024 Apr 8;11:353. doi: 10.1038/s41597-024-03197-0 (PMC11002007; doi:10.1038/s41597-024-03197-0)
Supplement: Supplementary file 1 — Supplementary Information [file 41597_2024_3197_MOESM1_ESM.pdf]

Supplementary Information

Table of Contents

Supplementary Figure 1..*page 2*

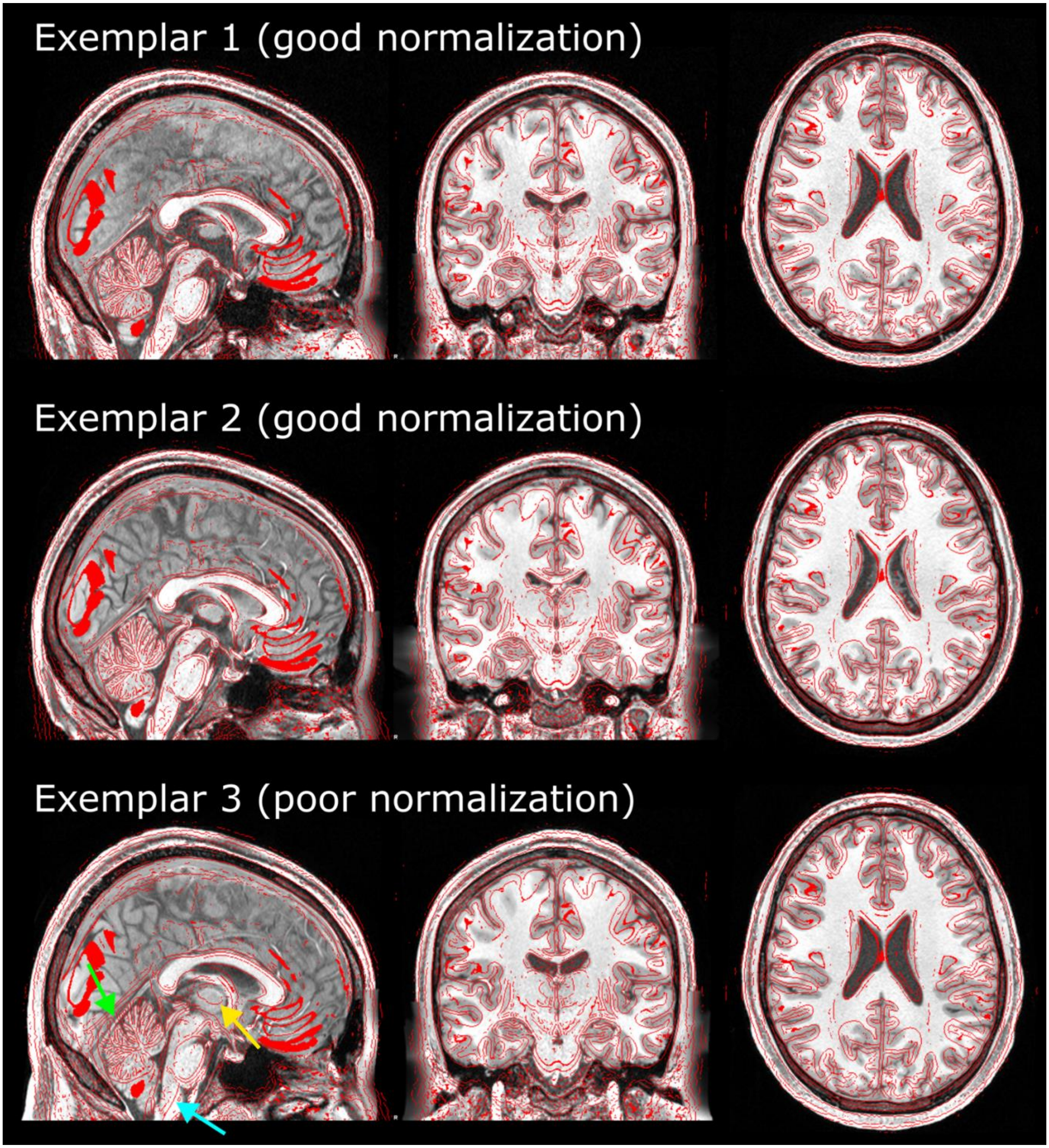

Supplementary Figure 1. Manual quality control of image registration. The adequacy of each coregistration and normalization transformation was manually assessed by examining quality control images generated by the Lead-DBS software package (https://www.lead-dbs.org/). These images showed sagittal, coronal, and axial slices of the transformed source brain overlaid on the target brain. The contours of prominent brain structures, including the cerebellum, brainstem, thalamus, and

cerebral lobes, were carefully compared between the source and target images to ensure a close degree (i.e., within a few millimetres) of alignment. Exemplar images from the second round of multispectral normalization show three HCP subjects' transformed T1-weighted images superimposed on a red wire-frame structure denoting the MNI ICBM 2009b NLIN Asymmetric template. Exemplars 1 and 2 show good normalizations, while Exemplar 3 depicts a poor normalization that was ultimately discarded. Arrows draw attention to specific brain regions where poor alignment between the source and target images was observed (green: cerebellum and occipital lobe; turquoise: brainstem; yellow: thalamus). *HCP* = Human Connectome Project; *MNI* = Montreal Neurological Institute.
